# Subclinical lipopolysaccharide from *Salmonella* Enteritidis induces neuropeptide dysregulation in the spinal cord and the dorsal root ganglia

**DOI:** 10.1186/s12868-019-0502-z

**Published:** 2019-04-25

**Authors:** Anita Mikołajczyk, Dagmara Złotkowska

**Affiliations:** 1 Department of Public Health, Faculty of Health Sciences, Collegium Medicum, University of Warmia and Mazury in Olsztyn, 10-082 Olsztyn, Poland; 2 Department of Food Immunology and Microbiology, Institute of Animal Reproduction and Food Research, Polish Academy of Sciences in Olsztyn, 10-748 Olsztyn, Poland

**Keywords:** LPS from *S.* Enteritidis, Neuropeptides, Dorsal root ganglia (DRG), Spinal cord, Substance P (SP), Galanin (GAL), Neuropeptide Y (NPY), Vasoactive intestinal peptide (VIP), Somatostatin (SOM)

## Abstract

**Background:**

Despite increasing evidence that lipopolysaccharide (LPS) affects the biological active substances of dorsal root ganglia (DRG) we have limited knowledge of the influence of a single low dose of LPS, which does not result in any clinical symptoms of disease (subclinical LPS) on neuropeptides connected with the sensory pathway. Accordingly, in this work, we investigated the influence of subclinical LPS from *Salmonella* Enteritidis on selected neuropeptides: substance P (SP), galanin (GAL), neuropeptide Y (NPY), vasoactive intestinal peptide (VIP) and somatostatin (SOM) in the cervical, thoracic, lumbar and sacral regions of the DRG and spinal cord.

**Methods:**

This study was performed on immature female pigs of the Pietrain × Duroc breed. Seven days after the intravenous injection of saline solution for control animals (n = 5) and 5 μg/kg b.w. LPS from *S.* Enteritidis for the experimental group (n = 5), the DRG and the spinal cord were collected to extract the neuropeptides using solid-phase extraction technology.

**Results:**

Our results demonstrated that subclinical LPS in DRG was able to change the levels of all studied neuropeptides except SOM, whereas in the spinal cord it down-regulated all studied neuropeptides in the sacral spinal cord, maintaining the concentration of all studied neuropeptides in other regions similar to that observed in the control animals. The significant differences in the intensity and character of observed changes between particular regions of the DRG suggest that the exact functions of the studied neuropeptides and mechanisms of responses to subclinical LPS action depend on specific characteristics and functions of each examination region of DRG.

**Conclusions:**

The mechanisms of observed changes are not fully understood and require further study of the molecular interactions between subclinical LPS from *S*. Enteritidis and neuronal and non-neuronal cells of DRG and spinal cord. The peripheral and central pain pathways must be analysed with the aspect of unknown long-term consequences of the influence of subclinical LPS from *S.* Enteritidis on neuropeptides in the spinal cord and the dorsal root ganglia.

## Introduction

Dorsal root ganglia (DRG), with their cell bodies of sensory (afferent) neurons, play an essential role in the transduction of the sensory and pain signals from the periphery to the spinal cord and onward to the brain [1]. Pathological conditions such as inflammation and nerve injury can sensitize DRG neurons and change their neurochemical characterization. Changes in the neuropeptides, such as substance P (SP), galanin (GAL), neuropeptide Y (NPY), vasoactive intestinal peptide (VIP) and somatostatin (SOM) in DRG and spinal cord are associated with pain pathways, the mechanisms of pathological processes and potential therapeutic strategy development or increase the trophic support of some chronic diseases [2–6].

Sensory symptoms, especially pain, sensory disturbances and dysfunction of the autonomic nervous system are characteristic features of patients with Parkinson’s disease (PD) and in other synucleinopathies caused by the abnormal accumulation of a-synuclein in neurons, glia or both [7–10]. The abnormal accumulation of pathologic α-synuclein during PD takes place in the central and peripheral nervous systems, including the spinal cord and DRG of PD patients [11]. Perrotta et al. [12] suggested that in the preclinical stages of PD and in early-stage of PD with the absence of clinical pain syndrome, the facilitation of pain processing may be driven not only by dopaminergic differentiation but also by degenerative processes modulating the spinal cord. Since the problem of pain and sensory disturbances in neurodegenerative disorders is critically important, a comprehensive understanding of mechanisms and predisposing factors is still necessary [8].

There is a growing body of evidence that inflammatory triggers such as lipopolysaccharide (LPS) may be involved in the neurodegenerative processes and the sensory pathways connected with them [13]. A number of studies have used LPS animal models for PD [14], for systemic inflammation [15], sepsis [16] and for inducing neuroinflammation, which is an important feature in neurodegenerative diseases such as Alzheimer´s disease, PD and amyotrophic lateral sclerosis [17]. A high dose of LPS, administered usually directly into the substantia nigra, has been for years used in experimental animal models mimicking the symptoms of Parkinson’s disease in people [13, 14, 18, 19]. These models make use of the LPS ability to activate microglia cells to release the inflammatory mediators. LPS is an endotoxin found on the outer membrane of pathogens and non-pathogen gram-negative bacteria. Lipopolysaccharides are molecules composed of lipids and polysaccharides and, although these can have virulent properties, their function for the bacteria is primarily structural [20, 21]. LPS shows structural differences not only between bacterial species but also within particular serotypes [22]. Our previous in vitro observations showed that structural different serotypes of LPS from *Salmonella* spp. result in a varied impact on the nervous system. Changes in immunoreactivity to neuropeptides of DRG neurons clearly depended on bacterial serotype, for example, a low dose of *S.* Enteritidis caused a decrease in the number of SP-positive DRG neurons, whereas the same dose of LPS but from *S.* Minnesota or from *S.* Typhimurium resulted in a decrease in the percentage of such cells. In contrast to LPS from *S.* Enteritidis and *S.* Minnesota, LPS from *S.* Typhimurium did not influence neuron immunoreactivity to GAL [23].

Moreover, the presence of LPS from pathogens such as *Salmonella* spp. in the body can last for years [24]. Despite current achievements, there are numerous difficulties in detecting LPS, not only in a live organism but also in drugs and biological products. Even critical standards of endotoxin detection have been established in many countries to regulate endotoxin limits in the mentioned products. The greatest limitation of the detection of LPS is associated with low sensitivity and the lack of the possibility of detecting serogroup–specific antigens [25, 26]. Additionally, asymptomatic *Salmonella* carrier state [27] and the long–unsolved problem of the transmission *Salmonella* spp. to humans from contaminated food [28–30] are among the most baffling of medical problems in public health and in epidemiology.

Taking everything mentioned above into consideration, we decided to investigate the influence of asymptomatic *S.* Enteritidis on levels of selected neuropeptides connected with sensory pathways (SP, GAL, NPY, VIP and SOM) in the cervical, thoracic, lumbar and sacral regions of the spinal cord and DRG of the domestic pig. It should be noted that the best biomedical model for investigating the pathogenesis and treatment of many human disease is the pig. In order to understand the processes occurring in the human body, the porcine model was selected for the study, as pigs are phylogenetically closer to people than mice or rodents and the studies on them are characterised by the repeatability of results. This species shows great similarity to the human body anatomically, physiologically and immunologically. Thus, the results obtained may accurately reflect the mechanisms connected with the effect of LPS on the human body. Additionally using an animal model made it possible to carry out studies, the conduction of which would be highly problematic in people, both from the ethical and technical point of view [31–34].

Furthermore, a feature of the DRG is its lack of the barriers, which in the spinal cord constrain the entry of substances from circulation. How LPS peripherally induces its effects on the spinal cord is unknown. However, LPS can stimulate the increase of permeability of blood-spinal cord barrier (BSCB) and the blood-cerebrospinal fluid barrier (BCSFB) and cause a significantly higher influx rate of cytokines from blood to the central nervous system [35, 36]. Thus, it is hypothesized that subclinical LPS from *S.* Enteritidis can modulate levels of selected neuropeptides (SP, GAL, NPY, VIP and SOM) both in the DRG and in the spinal cord.

## Results

Both the control and the experimental group of animals were without any symptoms of disease during every day of this investigation. Over the period of the experiment, there were no differences in health status, appearance, temperature or body weight between animals of the control and the LPS groups. In both animal groups, immunoreactivities for all studied neuropeptides were widely distributed within the DRG and in the spinal cord.

**Fig. 1:**
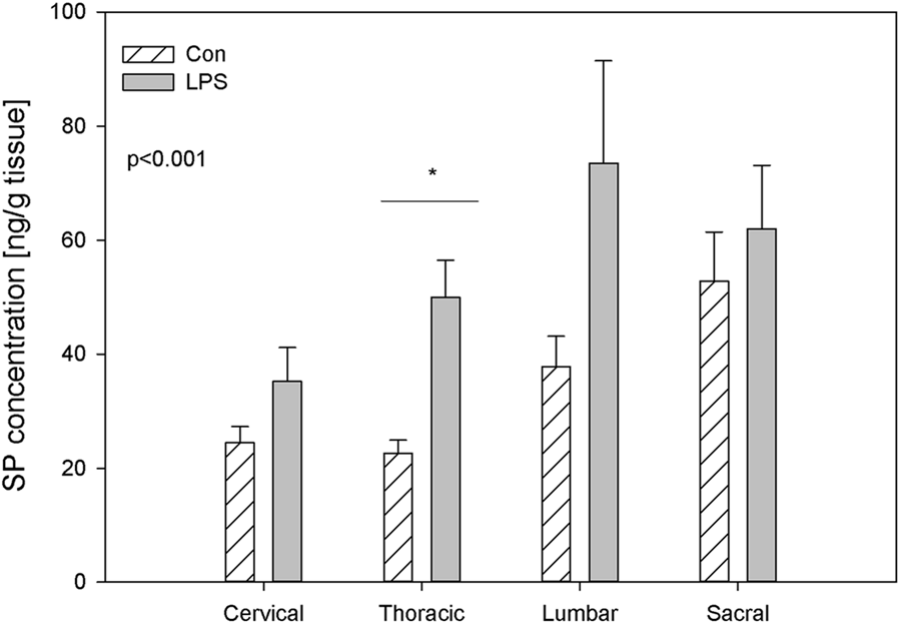
The average substance P (SP) content in the cervical, thoracic, lumbar and sacral dorsal root ganglia (DRG) of control pigs (Con, n = 5) and the LPS-treated group (LPS, n = 5). The values are presented as the average from group ± SD. The data were statistically analysed using one-way ANOVA and subsequent comparisons within groups were performed using Tukey’s test. *statistically different for p < 0.001

**Fig. 2:**
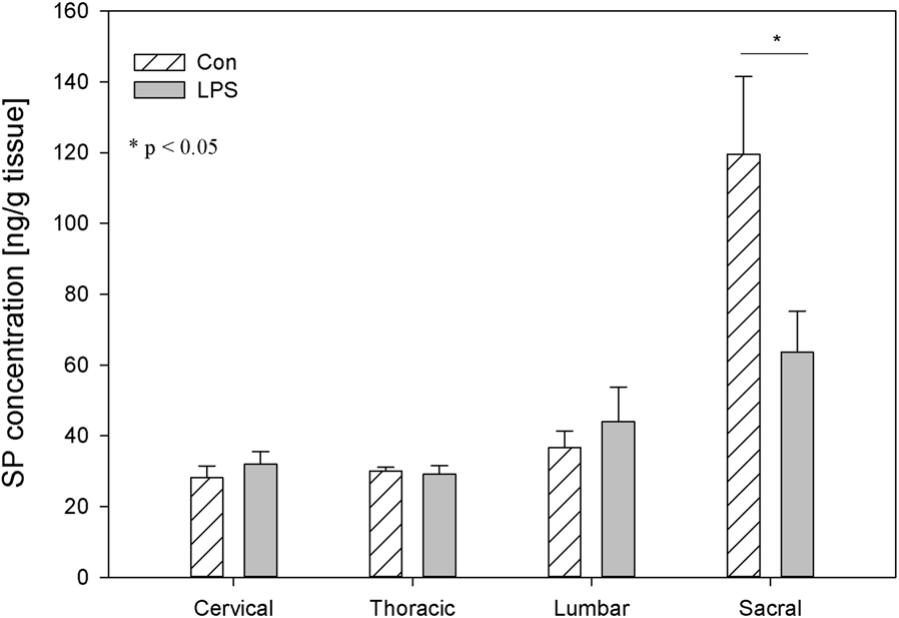
The average substance P (SP) content in the cervical, thoracic, lumbar and sacral spinal cord of control pigs (Con, n = 5) and the LPS-treated group (LPS, n = 5). The values are presented as the average from the group ± SD. The data were statistically analysed using one-way ANOVA and subsequent comparisons within groups were performed using Tukey’s test. *statistically different for p < 0.05

The highest concentration in both DRG and in the spinal cord was observed for SP. In the control animals, the level of SP exceeded 20 ng/g tissue (from 22.64 ± 2.29 ng/g tissue in the thoracic DRG to 52.79 ± 8.65 ng/g tissue in the sacral DRG and from 28.16 ± 3.33 ng/g tissue in the cervical spinal cord to 119.56 ± 21.99 in the sacral spinal cord) in all investigated regions of DRG and the spinal cord (Figs. 1, 2). LPS administration caused significant changes in SP levels only in the thoracic DRG (the increase from 22.64 ± 2.29 ng/g tissue to 50.00 ± 6.49 ng/g tissue) and in the sacral spinal cord (the decrease was from 119.56 ± 21.99 ng/g tissue to 63.61 ± 11.63 ng/g tissue). Except for changes of concentration of SP in thoracic DRG and in the sacral spinal cord, LPS compared to the control did not statistically significantly change the SP levels in the other investigated regions of the DRG and the spinal cord (Figs. 1, 2).

**Fig. 3:**
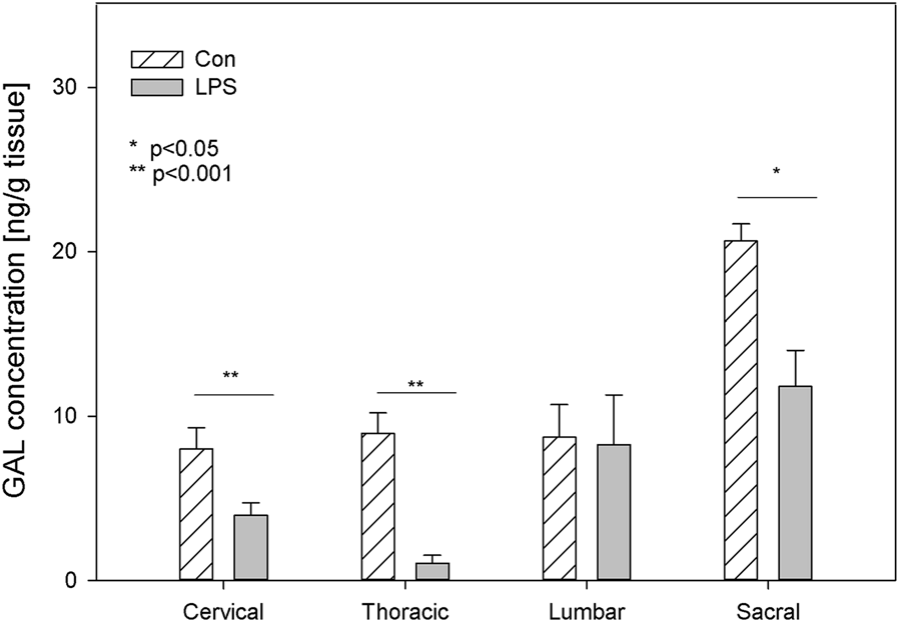
The average galanin (GAL) content in the cervical, thoracic, lumbar and sacral dorsal root ganglia (DRG) of control pigs (Con, n = 5) and the LPS-treated group (LPS, n = 5). The values are presented as the average from group ± SD. Data were statistically compared using one-way ANOVA and subsequent comparisons within groups were performed using Tukey’s test. *statistically different for p < 0.05, **statistically different for p < 0.001

**Fig. 4:**
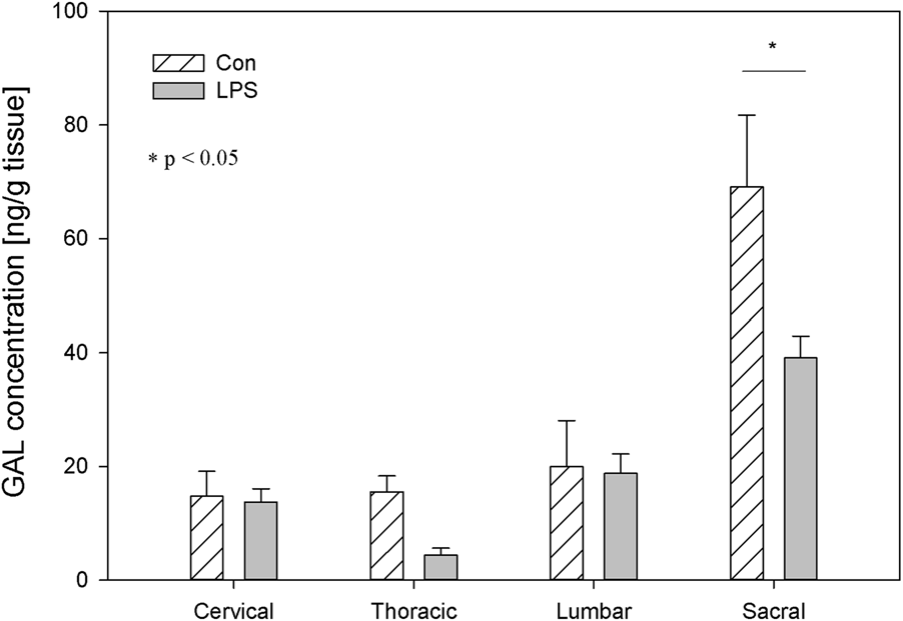
The average galanin (GAL) content in the cervical, thoracic, lumbar and sacral spinal cord of control pigs (Con, n = 5) and the LPS-treated group (LPS, n = 5). The values are presented as average from group ± SD. Data were statistically compared using one-way ANOVA and subsequent comparisons within groups were performed using Tukey’s test. *statistically different for p < 0.05

GAL was a substance whose level during the present study in control animals was lower than the concentration of SP, but higher than the concentration of other examined neuropeptides. In the DRG of control animals, the concentration of this substance ranged from 8.02 ± 1.29 ng/g tissue in the cervical ganglia to 20.65 ± 1.04 ng/g tissue in the sacral DRG (Fig. 3). In the spinal cord of control animals, the concentration of GAL fluctuated from 14.76 ± 4.39 ng/g tissue in the cervical region to 69.08 ± 12.65 ng/g tissue within the sacral spinal cord (Fig. 4). LPS administration induced a decrease in GAL levels in all parts of the DRG except lumbar DRG, e.g. in the cervical DRG (from 8.02 ± 1.29 ng/g tissue to 3.96 ± 0.77 ng/g tissue), thoracic DRG (from 8.96 ± 1.24 ng/g tissue to 1.07 ± 0.47 ng/g tissue) and sacral DRG (from 20.65 ± 1.04 ng/g tissue to 11.82 ± 2.20 ng/g tissue) (Fig. 3). A subclinical dose of LPS statistically significantly changed the concentration of spinal cord GAL only in the sacral spinal cord, where LPS administration induced a decrease in GAL concentration from 69.08 ± 12.65 ng/g tissue to 39.09 ± 3.81 ng/g tissue (Fig. 4).

**Fig. 5:**
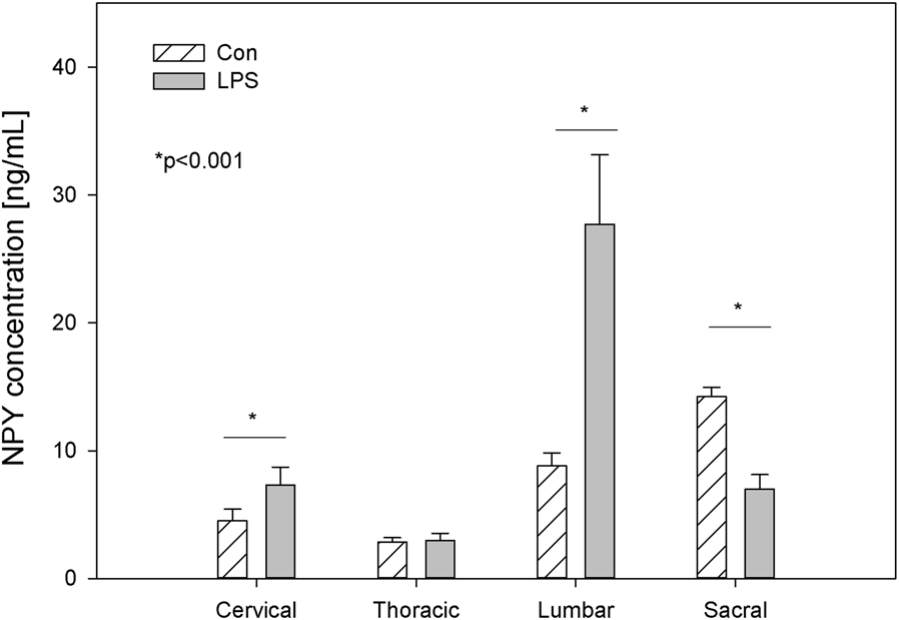
The average neuropeptide Y (NPY) content in the cervical, thoracic, lumbar and sacral dorsal root ganglia (DRG) of control pigs (Con, n = 5) and the LPS-treated group (LPS, n = 5). The values are presented as average from group ± SD. Data were statistically compared using one-way ANOVA and subsequent comparisons within groups were performed using Tukey’s test. *statistically different for p < 0.001

**Fig. 6:**
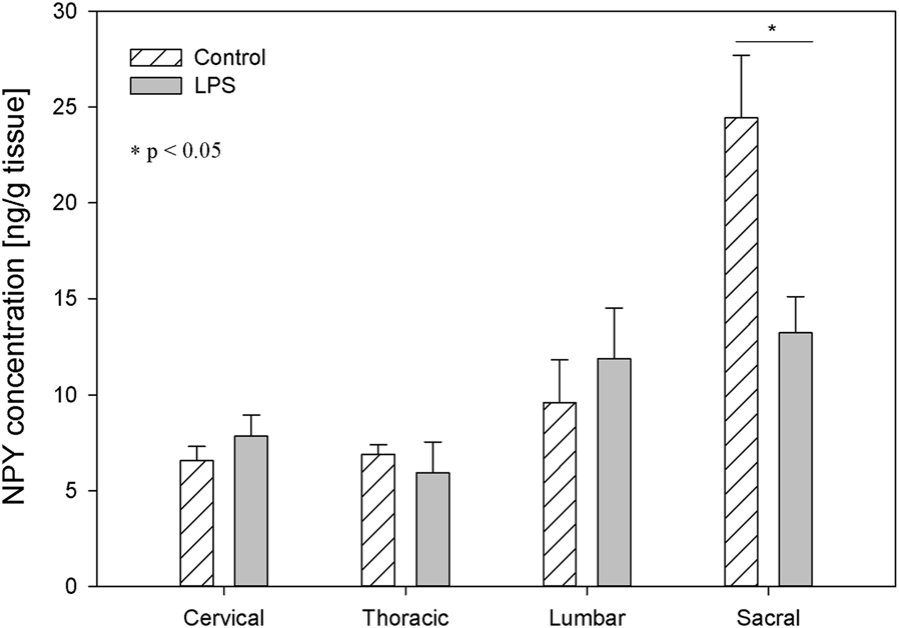
The average neuropeptide Y (NPY) content in the cervical, thoracic, lumbar and sacral spinal cord of control pigs (Con, n = 5) and the LPS-treated group (LPS, n = 5). The values are presented as average from group ± SD. Data were statistically compared using one-way ANOVA and subsequent comparisons within groups were performed using Tukey’s test. *statistically different for p < 0.05

NPY is a biological active substance whose level underwent LPS–induced changes in relatively numerous parts of the DRG. In the DRG of control animals, the concentration of this substance fluctuated from 2.85 ± 0.34 ng/g tissue in the thoracic DRG to 14.23 ± 0.73 ng/g tissue within the sacral DRG (Fig. 5). The level of NPY in the spinal cord of control animals ranged from 6.57 ± 0.74 ng/g tissue in the cervical region to 24.43 ± 3.27 ng/g tissue in the sacral spinal cord (Fig. 6). LPS administration changed the concentration of NPY in all parts of DRG, except the thoracic DRG. The character and intensity of changes clearly depended on the investigated regions of DRG. In particular, an increase in the NPY concentration after LPS administration was observed within the cervical and lumbar DRG (from 4.50 ± 0.93 to 7.33 ± 1.39 ng/g tissue and from 8.83 ± 1.00 to 27.72 ± 5.45 ng/g tissue respectively), whereas in sacral DRG, the level of this substance was clearly lower and decreased from 14.23 ± 0.73 ng/g tissue in the control group to 7.00 ± 1.13 ng/g tissue in the LPS group (Fig. 5). An almost two fold decrease was observed in the sacral spinal cord NPY concentration, from 24.43 ± 3.27 ng/g tissue to 13.25 ± 1.86 ng/g tissue after LPS induction. (Fig. 6).

**Fig. 7:**
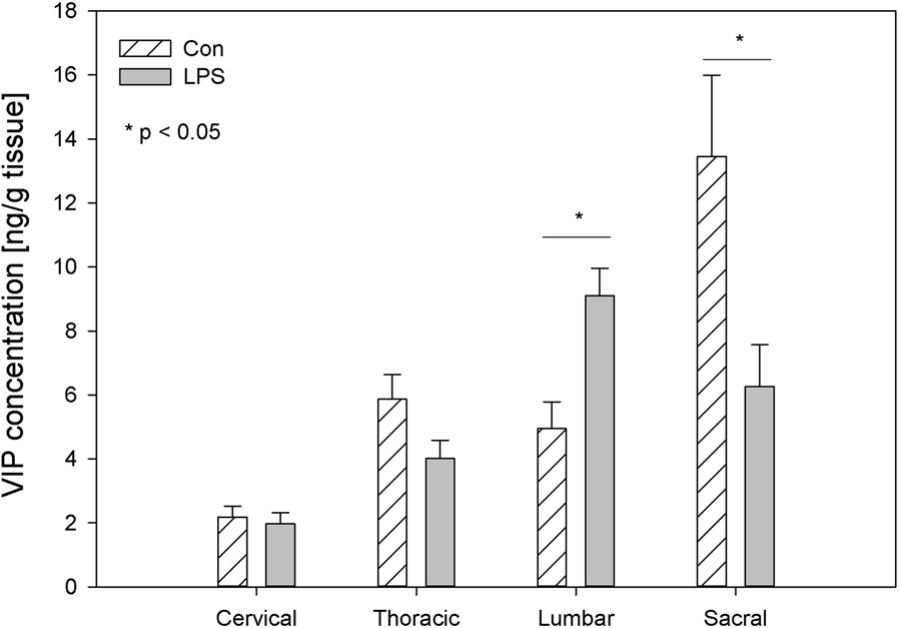
The average vasoactive intestinal peptide (VIP) content in the cervical, thoracic, lumbar and sacral dorsal root ganglia (DRG) of control pigs (Con, n = 5) and the LPS-treated group (LPS, n = 5). The values are presented as average from group ± SD. Data were statistically compared using one-way ANOVA and subsequent comparisons within groups were performed using Tukey’s test. *statistically different for p < 0.05

**Fig. 8:**
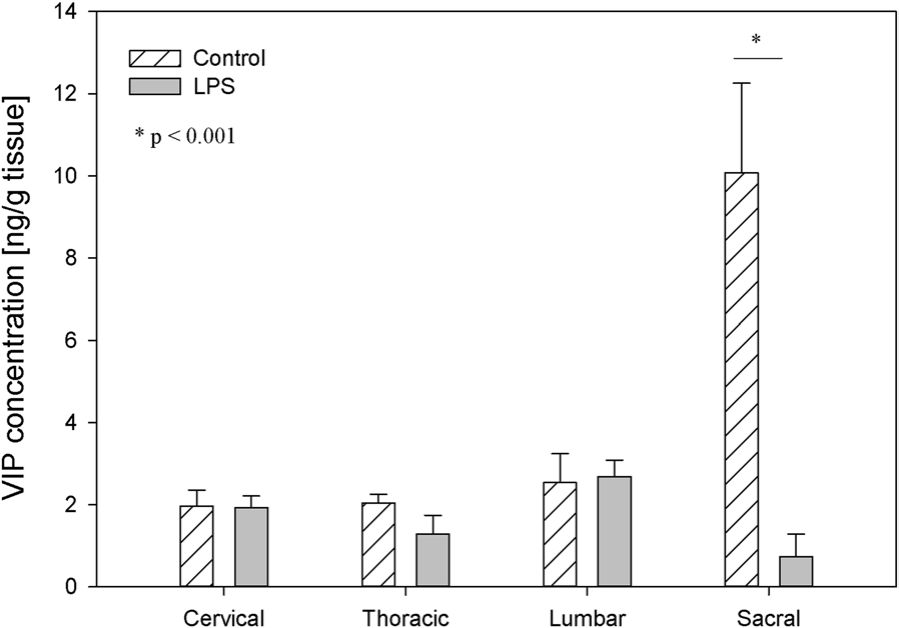
The average vasoactive intestinal peptide (VIP) content in the cervical, thoracic, lumbar and sacral spinal cord of control pigs (Con, n = 5) and the LPS-treated group (LPS, n = 5). The values were statistically compared using one-way ANOVA and subsequent comparisons within groups were performed using Tukey’s test. *statistically different for p < 0.001

In the control animals, the levels of VIP fluctuated in DRG from 2.18 ± 0.35 ng/g tissue in the cervical ganglia to 13.45 ± 2.54 ng/g tissue in the sacral DRG, and in the spinal cord from 1.98 ± 0.39 in the cervical ganglia to 10.08 ± 0.74 in the sacral DRG, respectively (Figs. 7, 8). LPS administration caused changes in VIP concentration within the lumbar and sacral DRG and in the sacral spinal cord. These changes were manifested by an increase within the lumbar DRG (from 4.95 ± 0.84 ng/g tissue to 9.10 ± 0.87 ng/g tissue), and a decrease in the sacral DRG (from 13.45 ± 2.54 ng/g tissue to 6.26 ± 1.31 ng/g tissue) and the sacral spinal cord (from 10.08 ± 0.74 ng/g tissue to 4.99 ± 0.55 ng/g tissue) (Figs. 7, 8).

**Fig. 9:**
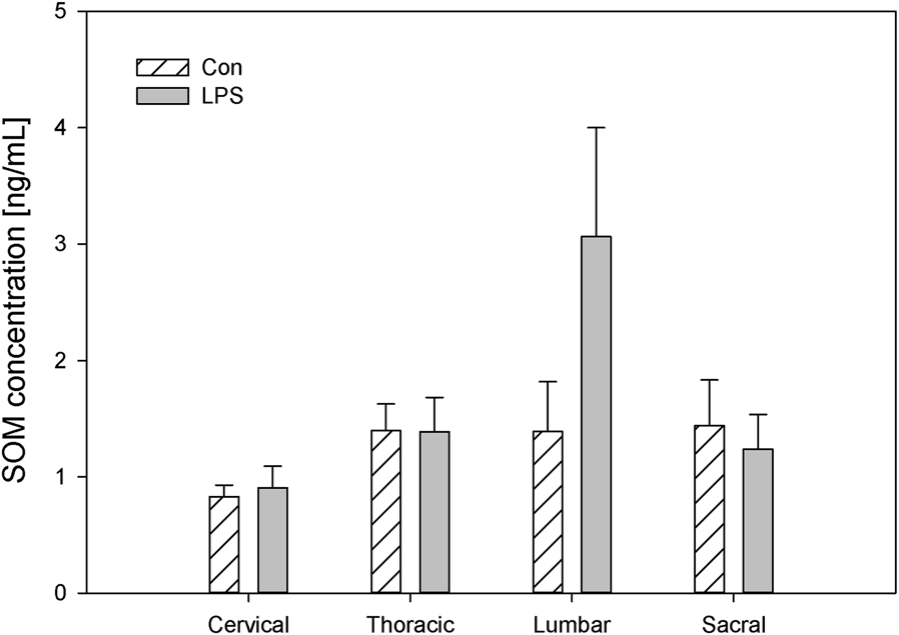
The average somatostatin (SOM) content in the cervical, thoracic, lumbar and sacral dorsal root ganglia (DRG) of control pigs (Con, n = 5) and the LPS-treated group (LPS, n = 5). The values are presented as average from group ± SD. Data were statistically compared using one-way ANOVA and subsequent comparisons within groups were performed using Tukey’s test

**Fig. 10:**
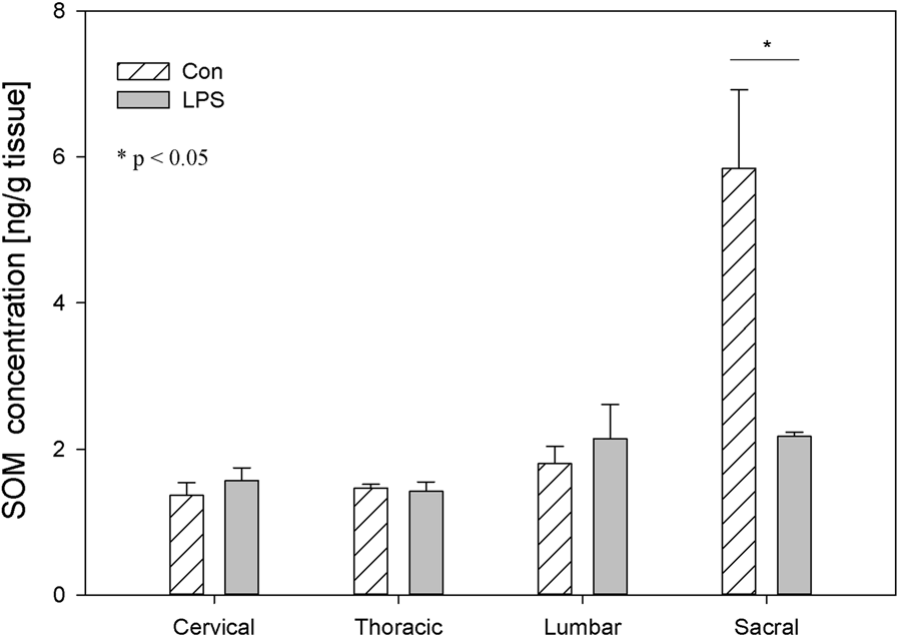
The average somatostatin (SOM) content in the cervical, thoracic, lumbar and sacral spinal cord of control pigs (Con, n = 5) and the LPS-treated group (LPS, n = 5). The values are presented as average from group ± SD. Data were statistically compared using one-way ANOVA and subsequent comparisons within groups were performed using Tukey’s test. *statistically different for p < 0.05

Both in the DRG and in the spinal cord, the levels of SOM observed during the present study in control animals were the lowest among the studied biological active substance and ranged in DRG from 0.83 ± 0.10 ng/g tissue in the cervical DRG to 1.44 ± 0.39 ng/g tissue within the sacral DRG and in spinal cord from 1.37 ± 0.17 ng/g tissue in the cervical region to 5.84 ± 1.08 ng/g tissue in the sacral spinal cord, respectively (Figs. 9, 10). Contrary to the other studied neuropeptides, subclinical LPS did not change the levels of SOM in the studied parts of DRG (Fig. 9). Only in sacral spinal cord was a significant decrease of SOM concentration observed (from 5.84 ± 1.08 ng/g tissue in the control group to 2.18 ± 0.06 ng/g tissue in the LPS group) (Figs. 9, 10).

## Discussion

Our study showed that a low single dose of LPS *S.* Enteritidis, which does not result in any clinical symptoms of disease (subclinical LPS) can change levels of SP, GAL, NPY, VIP and SOM in the spinal cord and DRG. These findings are in agreement with previous studies that demonstrated that even a single low dose of subclinical LPS *S.* Enteritidis modulated the main porcine enteric neuropeptides in guts and changed the number and chemical coding of intramural nerves within the porcine gallbladder wall and dysregulated the levels of CRH, GnRH, TRH, GAL, NPY, SOM, SP and VIP in selected clinically significant brain structures and in the endocrine glands of HPA, HPO, HPT axes. [37–40]. Additionally, our other studies [41] showed that seven days after subclinical LPS *S*. Enteritidis administration, the study animals had significantly elevated haptoglobin (Hp) levels in the blood serum, but no statistically significant changes were observed in interleukin 6 (IL-6) and tumour necrosis factor α (TNF-α) serum levels between the LPS and the control groups.

It should be pointed that this study used solid-phase extraction (SPE) technology and enzyme immunoassays for the quantitative determination of neuropeptides in tissue extracts to measure the total content of neuropeptides, i.e. concentrations of neuropeptides located in the neurons, a fraction already released from neurons into the tissue and neuropeptides of non-neuronal origin. Among the non-neuronal cells that synthesize neuropeptides are several glial cell types. There is strong evidence that many neuropeptides are expressed in glia. SOM, SP, VIP GAL, NPY are especially released by astrocytes but also by microglia, Schwann cell precursors and oligodendrocytes precursors [42–44]. In many cases, glial neuropeptide levels are very low, but hypothetically during pathological processes, levels of glial neuropeptides can be changed [42]. Glial exocytosis in neuroinflammation is probably involved in the secretion of a wide variety of neuropeptides by glial cells. Despite gial exocytosis is much slower than its neuronal counterpart it can be involved in maintaining the homeostasis of neural network components [45]. However, it is known that glial cells can proliferate and more biological active substance can then be released into tissue. Glia plays a critical role in many pathological processes, starting with neurodegenerative disease and ending with joint inflammatory diseases. In chronic pain in joint inflammatory diseases, a critical role is played by the satellite glial cells (SGCs) that surround the cell bodies of primary afferent neurons in the DRG. A recent study indicated that SGCs activation particularly occurs after day 7 of arthritis and is involved in the mechanisms of articular inflammation [46]. In our study, neuropeptide levels were measured seven days after the administration of a single, subclinical LPS dose. A 7-day period is sufficient for the emergence of changes in the nervous system, which was also confirmed in previous studies [37–41]. Additionally, different types of non-neuron cells, such as SGCs in DRG, oligodendrocytes, astrocytes, microglia, in the spinal cord as well as immune cells (for example, macrophages and T-cells), release neuromodulatory substances in close proximity to neuronal cells, which either promote or dampen pain depending on the specific identities of the mediators involved [47]. Neuropeptides among other different active substances, can contribute to promoting or dampening inflammatory pain. Moreover, an increased release of some neuropeptides activates immune cells, thereby inducing a positive feedback of inflammation [48]. DRG and spinal cord not only respond to immune signals but can also directly modulate inflammation–releasing neuropeptides to dampen and constrain the immune response.

Until recently, it was generally believed that pathogens during infections active sensory neurons to cause pain through influence on the immune cells and the inflammatory substances. Chiu et al. [49] revealed that bacteria can directly stimulate sensory neurons to produce pain and suppress inflammation. Various bacteria use their components and mechanisms differently to activate sensory neurons. LPS potently induces inflammatory pain, a mechanism dependent on TLR4/MyD88 signalling [50]. LPS activates the sensory nervous system, produces pain hypersensitivity by sensitizing TRPV1 and TRPA1 in a TLR4-independent manner in sensory neurons [51]. The role that neuropeptides play in the body is very complex and, besides the different impact of neuropeptides, *inter alia* on neurodegenerative and neuroprotective processes, some neuropeptides themselves may have antimicrobial functions [52]. The mechanism of neuropeptide antimicrobial activity is still unclear and requires future studies to clarify whether the neuropeptides have direct or only indirect antimicrobial effects [53]. The neuropeptides SP, NPY, VIP have antimicrobial activity against a range of pathogens [54]. The antimicrobial activity of NPY is different in regard to various pathogens [55]. Gibran et al. [56] hypothesized that reduced neuropeptide levels may contribute to the delayed wound healing common in patients with diabetes mellitus. The importance of the neurogenic protection provided by SP can be appreciated in the sensory neuropathy during diabetes. SP applied to the wounds speeds the healing process in the diabetic animals [56].

The expression of SP in DRG neurons increases after peripheral inflammation and decreases after peripheral nerve injury [57]. Similarly, articular inflammation is known to increase SP expression in the DRG neurons [58] but osteoarthritis caused a decrease in SP in lumbar DRG [3]. In the present study, subclinical LPS did not exhibit SP up-regulation in cervical, lumbar and sacral DRG, maintaining SP concentration levels to those observed in control animals, but in thoracic DRG, there was a statistically significant decrease in SP levels in the LPS group compared to the control (Fig. 1). Our findings are in agreement with our previous in vitro study, where the low dose of LPS *S.* Enteritidis caused an increase in the number of SP-positive neurons in thoracic-lumbar DRG [23].

Another example of a neuropeptide involved in inflammation processes and pain signalling during spinal cord injury is GAL [59, 60]. Except for up-regulation in the levels of GAL in a variety of nerve injuries, GAL plays a neuroprotective role in experimental inflammatory demyelination in mice [61]. The presence of GAL and its receptors in DRG and the spinal cord creates opportunities to use GAL or agonists of its receptors to treat neuropathic pain [62]. GAL plays an important role in the development and regeneration of DRG sensory neurons, The elevated GAL levels in DRG neurons could promote trophic processes after injuries [63]. Considering the trophic role of GAL, unfortunately, we did not observe its growth in any of the regions of the DRG or spinal cord in the LPS group of our study (Figs. 3, 4). To the contrary, we observed a statistically significant decrease in GAL levels in the sacral spinal cord, cervical, thoracic and sacral DRG in the LPS group compared to control (Figs. 3, 4). Similar to our previous in vitro study, the low dose of LPS *S.* Enteritidis caused a decrease in the number of GAL-positive neurons in thoracic-lumbar DRG [23].

Similarly to GAL [62, 64], NPY [65, 66] is also involved in the modulation of neuropathic pain induced by peripheral nerve injury. While both nociceptive and antinociceptive effects of NPY have been described, it is generally believed that this peptide is mainly antinociceptive. [67]. NPY is synthesized in the DRG and the spinal cord, where it inhibits the nociceptive pathway, serving as an adaptive compensatory mechanism in response to excessive excitatory signalling [68]. NPY exerts antinociceptive and analgesic effects by inhibiting the release of SP in the spinal cord dorsal horn and activating Y1 spinal receptors both during and without inflammatory nociception [65, 69, 70]. Moreover, NPY was shown to inhibit the release of SP from DRG neurons [71]. Following peripheral nerve injury, NPY is dramatically up-regulated in the sensory ganglia of the peripheral nervous system [72]. It is known that an increase of NPY in the DRG and the spinal cord is followed after peripheral nerve injury and osteoarthritis but not after painful inflammation [3, 73–75]. Similar to after painful inflammation, in our study subclinical LPS induced a decrease in NPY in sacral DRG and the sacral spinal cord (Figs. 5, 6). In turn, in cervical and in lumbar DRG, we observed an increase of NPY (Fig. 5). Similarly, in lumbar DRG Adaes et al. [3] observed the increase in NPY expression in DRG neurons during osteoarthritis. Such NPY up-regulation may be a feedback mechanism to counteract the subclinical inflammatory process since NPY is, in turn, able to inhibit the release of inflammatory substances via activation of Y1 receptors [76]. Since microglial and astroglial cells present NPY receptors [43, 76], hypothetically NPY can act directly in glial cells by regulating their action to reduce neuronal dysfunction. However, it is also possible that the anti-inflammatory effects of NPY and a reduction of neuronal dysfunction result from other NPY-mediated protective mechanisms and might be connected with the NPY role in decreasing the toxic stimulus that triggers glia activation and dysfunction of neurons. Therefore, NPY can reduce neuroinflammation and this effect might mediate neuroprotection.

Since NPY and VIP have crucial trophic effects that are critical for joint tissue and bone homeostasis, it appears increasingly likely that those neuropeptides or their respective receptor agonists/antagonists may be exploited for the treatment of patients with pain and/or inflammatory and/or degenerative joint diseases in the future [6]. Antagonists that inhibit VIP activity may prove beneficial in the alleviation of osteoarthritis pain. Xiao et al. [77] observed that the score of pain intensity during osteoarthritis and osteoporosis was correlated positively with the average of the optical density values for SP, and VIP and correlated negatively with values for NPY [78]. VIP and its receptors present in the DRG and spinal cord contribute to the altered transmission of sensory information in neuropathic pain conditions [79]. VIP, GAL and SP are mediators in visceral pain. A short-term noxious mechanical distension of the rectum changed their levels in the spinal cord in rats [80]. VIP is over-expressed in DRG neurons after injury and the peripheral sensory nervous system played a crucial role in the in vitro model of human skin wound healing. In that model, DRGs neurons and VIP and SP stimulated skin cell proliferation [81]. On the other hand, the protective role of VIP in inflammatory diseases causes that even in LPS-induced shock, VIP decrease cytokine levels and mortality in mice with exogenous administration [82]. The subclinical LPS used in our study induced an increase in VIP level only in lumbar DRG (Fig. 7), which may hypothetically be connected with the subclinical pro-inflammatory activity of the low LPS dose used in our study.

SOM is like other studied neuropeptides expressed in both the central and peripheral nervous systems and is involved in the regulation of several physiological and pathological processes. Both in central and in the peripheral nervous system, SOM can exert an analgesic effect. SOM released into circulation from capsaicin-sensitive afferents in chronic inflammation (arthritis) exerts systemic anti-inflammatory and analgesic effects [83]. SOM and their analogues have been described which are particularly interesting in terms of drug development. The fact that SOM is released into circulation from the activated TRPV1-expressing nociceptors (LPS also activates the sensory nervous system by sensitizing TRPV1) and the presence of somatostatin receptor type 4 in the DRG and in spinal cord cause the possible occurrence of a novel potential drug on even such excruciating pain as bone cancer pain [84, 85]. However, on the other hand, there was also conflicting evidence that SOM might contribute to nociception. Prasoon et al. [86] observed an increased immunoreactivity of SOM in the lumbar spinal cord after post-incisional pain and suggested that SOM may contribute to post-incisional pain. SOM has a dual effect in spinal nociceptive processing dependent on various kinds of noxious stimuli. SOM may suppress the responses of dorsal horn neurons to noxious heat and mechanical stimuli or may facilitate the responses of dorsal horn neurons to noxious cold stimuli [87]. Although SOM plays an important role in spinal nociceptive processing, knowledge of its activity is too limited to explain why subclinical LPS in the present study did not affect SOM levels in DRG but caused a decreased concentration of SOM in the sacral spinal cord (Figs. 9, 10). Similarly, other studied neuropeptides decreased their levels in the sacral spinal cord in LPS group compared to the control (Figs. 2, 4, 6, 8, 10).

It should be pointed that our results demonstrate down-regulation of all studied peptides in the sacral spinal cord after subclinical LPS (Figs. 2, 4, 6, 8, 10). Moreover, it is interesting that in the sacral DRG, down-regulation of all neuropeptides except SP and SOM was observed (Figs. 1, 3, 5, 7, 9). Perhaps the lack of change in SP and SOM levels in DRG in the LPS group compared to the control is caused by the interaction between the studied neuropeptides. During the activation of DRG cells, pro-nociceptive and pro-inflammatory neuropeptides (e.g. SP) are also released from them, which trigger a local neurogenic inflammation (local efferent function). Besides pro-inflammatory substances, analgesic and anti-inflammatory peptides (e.g. SOM) are also released, enter the systemic circulation and exert their effects on the whole body (systemic efferent or “sensocrine” function). Why subclinical LPS down-regulates all peptides in the sacral spinal cord is difficult to explain. The sacral autonomic outflow is spinal. Owing to its location, the parasympathetic system is commonly referred to as having “craniosacral outflow”, which stands in contrast to the sympathetic nervous system, which is said to have “thoracolumbar outflow”. It should be pointed out that the classical nomenclature of the sacral autonomic outflow has been recently challenged. Recent controversial papers [88, 89] have suggested that all sacral autonomic output may be sympathetic; indicating that the rectum, bladder and reproductive organs may only be innervated by the sympathetic nervous system. Jänig and Neuhuber [90] considered that the changes in the classification of the spinal autonomic nervous system proposed by Espinosa-Medina et al. [88] did not reflect the functional complexity of pelvic organ regulation. All of those references correctly argue that the transmitter status of autonomic neurons is very intricate and needs future study combining all fields of neuroscience [91].

## Conclusion

Considering our analyses and present results, the influence of subclinical LPS on neuropeptides must be analysed with the aspect of unknown long-term consequences as a result of changes in neuropeptides levels, which were not observed in the control group. Therefore, ignoring knowledge of the influence of subclinical LPS on biological active substances can limit our understanding of host–pathogen biology and disease processes. In conclusion, LPS from *S.* Enteritidis can modulate levels of selected neuropeptides, both in the DRG and the spinal cord. Downregulation of all studied neuropeptides in the sacral spinal cord was found after subclinical LPS, maintaining the concentrations of all studied neuropeptides in cervical, thoracic and lumbar regions similar to those observed in the control animals. In the DRG subclinical LPS was able to change the levels of all studied neuropeptides except SOM. The significant differences in the intensity and character of observed changes between particular regions of the DRG suggest that the exact functions of the studied neuropeptides and mechanisms of responses to subclinical LPS action depend on specific characteristics and functions of each examination region of DRG. The mechanisms of observed changes are not fully understood and require further study of the molecular interactions between subclinical LPS from *S.* Enteritidis and neuronal and non-neuronal cells of DRG and spinal cord. The pain pathways at central and peripheral sites must be analysed with the aspect of unknown long-term consequences of the influence of subclinical LPS from *S.* Enteritidis on neuropeptides in the spinal cord and the dorsal root ganglia.

## Materials and methods

### Animals and experimental procedures

All experimental procedures used in the present study were conducted according to the guidelines of the Local Ethics Committee for Animal Experimentation in Olsztyn located at University of Warmia and Mazury in Olsztyn and affiliated with the National Ethics Commission for Animal Experimentation, Polish Ministry of Science and Higher Education (decision No. 73/2015 from 29th Sept 2015). Two weeks before the beginning of the experiment, the animals were transported from a commercial farm to the local animal facility where they were kept under standard laboratory conditions in accordance with the experimental animal use and welfare requirements set by the Federal Law of 15 January 2015 on Animal Welfare for Science and Education.

Ten immature female pigs (Pietrain × Duroc), aged 8–9 weeks and weighing 16–18 kg were used in the present study. The animals were clinically healthy with negative results of analyses of *Salmonella* in faecal samples. During the experiment, all animals were kept in typical laboratory conditions and fed with a commercial grain mixture and tap water ad libitum. All efforts were made to limit the number of animals used and their suffering.

After a two-week adaptive period, the clinically healthy pigs were randomly divided into two groups (5 pigs in each group): a control group (Con, n = 5) and an experimental group (LPS, n = 5) and subjected to premedication, according to the method previously described by Mikołajczyk [92] with intramuscular injection of atropine (Atropinum Sulfuricum Polfa Warszawa S.A., Poland, 0.035 mg/kg b.w.), ketamine (Bioketan, Vetoquinol Biowet Sp. z o.o., Poland & Vetoquinol S.A., France, 7.0 mg/kg b.w.) and medetomidine (Cepetor, CP-Pharma Handelsges mbH, Germany, 0.063 mg/kg b.w.).

Under premedication, the control animals were injected with 10 mL saline solution, while pigs of the LPS group received LPS from *Salmonella* enterica serotype Enteritidis (catalogue no. L7770 Sigma, Aldrich, Germany) at a dose of 5 ug/kg b.w. (in 10 ml saline solution). Such a dose has been previously described as a “low single, subclinical” dose, which does not result in any clinical symptoms of disease [37, 41]. Injections in control and experimental animals were performed in the same way, i.e. intravenously into the marginal ear vein. The veterinary surgeon (DVM, Ph.D.) was managed all procedures, administered all drugs and conducted a clinical assessment of the pigs’ health status every day of the experiment. The physical examination, the measurements of temperature and body weight, both in the control and LPS group and the observations of the animal care staff were always taken into account by the veterinary surgeon during a clinical assessment of the pigs’ health status and were previously described by Mikołajczyk and Złotkowska [41].

Seven days after LPS administration (sufficient time post injection to sample collection for the emergence of changes in the nervous system [37, 40, 93] all clinically healthy animals were premedicated (in the above-described manner) and anesthetized with propofol (Scanofol, NORBROOK, Northern Ireland, IRL.PN, 4,5 mg/kg b.w. given intravenously) and then euthanized with pentobarbital (Morbital - mix of pentobarbital sodium 133.3 mg/mL with pentobarbital 26,7 mg/mL, Biowet-Puławy Sp. z o.o, Puławy, Poland, 60–70 mg/kg b.w., given intravenously). After euthanasia, the left and right of the cervical, thoracic, lumbar, and sacral DRG and the cervical, thoracic, lumbar and sacral spinal cord were collected. During the period of DRG and spinal cord collection, the tissues were poured 0.9% NaCl. Immediately after collection, the samples were packed, frozen in liquid nitrogen and stored at − 80 °C until analysis.

### High-temperature extraction procedure

The extraction of neuropeptide from animal tissue were prepared according to Conlon procedure [94]. In brief, after weighing and cutting frozen tissues 10 ml of hot 1 M acetic acid was added per gram tissue and boiled for 5 min. Then the samples were then homogenized using Ultra Turax IKA T-25 (Jankel & Kunkel IKA, Germany) at RT for 5 min and centrifuged at 4 °C for 40 min at 4500×g (Eppendorf 5804).

### Solid-phase extraction (SPE) technology, concentration and lyophilisation

The supernatants were filtered through syringe filters without pre-filter (Millex-HV Filter, 0.45 µm, PVDF, Millipore) or syringe filters with a graduated glass fibre pre-filter (Millex-HPF HV Filter, 0.45 μm, PVDF, Millipore). The filtrates of the biological fluids were acidified by trifluoroacetic acid (TFA) (final concentration 0.1% vol/vol). In the SPE technology, depending on the type and size of the sample, Sep-Pak Plus Light Cartridge (130 mg of C18 sorbent per cartridge, Waters, Milford, MA), or Sep-Pak C18 Plus Short Cartridge (360 mg of C18 sorbent per cartridge, Waters, Milford, were used according to the producer’s protocol using a Baker Vacuum Manifold SPE-12G unit (J.T.Baker, Germany). The volume of eluate was reduced on a miVac centrifugal vacuum concentrator, model DNA-23050-800 with SpeedTrap (Genevac Limited, UK) for two hours. Then samples were lyophilized using an ALPHA 1-4 LSC freeze dryer (MARTIN CHRIST Gefriertrocknungsanlagen GmbH Germany) and stored at − 80 °C until analysis.

The chemicals used for extraction: glacial acetic acid (cat. no. 951503, J.T. Baker), trifluoroacetic acid –TFA (cat. no. 9470, J.T. Baker) and acetonitrile-LC–MS reagent (cat. no. 9821.1000, J.T. Baker) were of high purity grade—HPLC grade.

### Quantitative determination of neuropeptides in tissue extracts

Peninsula Laboratories International, Inc. Tests for Substance P (0–5 ng/mL; cat. no. S-1180), Galanin (0–10 ng/mL; cat. no. S-1210) were used for SP and GAL determination, respectively.

Phoenix Pharmaceuticals, Inc. Tests for Vasoactive Intestinal Peptide (0–25 ng/mL; cat. no. EK-064-16CE), Neuropeptide Y (0–100 ng/mL; cat. no. EK-049-03CE), Somatostatin-28 (0–25 ng/mL; cat. no. EK-060-14CE) were used for VIP, NPY and SOM determination, respectively.

Samples were diluted according to the protocols provided by the manufacturer of Enzyme Immunoassay Kits and assayed in duplicates. Absorbance was read at λ = 450 nm on Infinite 200 (Tecan). A four-parameter ELISA curve was prepared for each determined neuropeptide (an Excel sheet was provided by Peninsula Laboratories service). Each sample was assayed in duplicate and the peptide concentration was read from the curve. Peptide concentrations were recalculated for 1 g of the tissue and are presented as the mean from group ± SD per g of tissue

### Statistical analysis

The results were analysed statistically using a one-way analysis of variance (ANOVA) and the significance of differences between groups was determined using Tukey’s test at a significance level of p < 0.05. The data were expressed as mean values ± SD and the calculations were performed with SigmaPlot^®^ 12 (Systat Software Inc.)

## Abbreviations

DRG: dorsal root ganglia
GAL: galanin
LPS: lipopolysaccharide
NPY: neuropeptide Y
PD: Parkinson’s disease
SPE: solid-phase extraction
SOM: somatostatin
SP: substance P
VIP: vasoactive intestinal peptide
